# Interspike intervals as a discrete time series with history and randomness

**DOI:** 10.1186/1471-2202-15-S1-P195

**Published:** 2014-07-21

**Authors:** Sharon E Norman, Robert J Butera

**Affiliations:** 1 School of Electrical and Computer Engineering, Georgia Institute of Technology, Atlanta, GA 30332, USA; 2 Department of Biomedical Engineering, Georgia Institute of Technology, Atlanta, GA 30332, USA

## 

Neurons are fundamental components of neural systems. Organized neural activity is key for proper function at many scales, from small central pattern generating circuits to large populations of neurons. Neurons can show considerable variability in their spiking regularity; this variability is attributed to a number of different sources, including probabilistic synaptic processes and stochastic ion channel activity [1]. The role of variability in neural processes is complex: for example, more variability in an input signal may enhance the consistency of output spiking [2], but spike variability poses problems for systems or theories that require precise event timing, such as spike-timing dependent plasticity [3].

In this work, we characterized the variability of intrinsically active neurons over time and found that all cells studied shared certain features. We measured intracellularly from synaptically-isolated, endogenously spiking *Aplysia californica* invertebrate neurons for 2 or more hours in a temperature-controlled preparation to determine the regularity of interspike intervals. We determined that sequential ISIs show strong positive correlation with prior ISIs and this correlation remains high for many lag values, indicating that the ISI sequence shows history. In contrast, sequential values of the change in ISI (delta ISI) show a single prominent negative autocorrelation coefficient; this indicates that delta ISI correlates strongest with one previous delta ISI, and the negative sign suggests a tendency for delta ISI values to remain within some range. We find that ISI sequences from intrinsically spiking neurons in this system can be represented using autoregressive integrated moving average (ARIMA) models. In addition, delta ISI distributions are symmetric and Gaussian-like with heavy tails. Recent literature reports that heavy-tailed ISI distributions that show positive serial correlation values may result from stochastic activity in populations of adapting ion channels [4,5]; we are currently investigating the cellular features that can produce an ARIMA process, paying particular attention to channel dynamics.

We present here an ISI variability model that captures statistical features of experimental data, which are essentially history and randomness. Existing explanations for neural spiking variability include properties of synaptic conductances [6], neural input [7], and ion channels [8]; these factors are modeled in simulation as or in combination with random processes that represent noisy inputs, which are then added to the membrane voltage. The phenomenological description of the ISI sequence itself as an ARIMA process with stochastic noise and history is a unique addition to the collection of neural noise models; it does not require explicit description or addition of cellular-level processes, it constrains correlational features rather than ISI distribution shape, and it represents intrinsic spike variability that is not due to synapses.

This work was funded by NIH NS 54281.
